# Early results after aortic annuloplasty with a complete external Dacron band

**DOI:** 10.1007/s11748-021-01695-1

**Published:** 2021-09-20

**Authors:** John-Peder Escobar Kvitting, Jan Otto Beitnes, Runar Lundblad

**Affiliations:** 1 Department of Cardiothoracic Surgery, Oslo University Hospital, Rikshospitalet, Nydalen, Post Office Box 4950, 0424 Oslo, Norway; 2 Department of Cardiology, Oslo University Hospital, Rikshospitalet, Oslo, Norway

**Keywords:** Aortic valve repair, External aortic annuloplasty, Bicuspid aortic valve

## Abstract

**Objective:**

This study evaluates the early results of our initial experience with aortic annuloplasty using a complete external Dacron band in the setting of type Ic or type II aortic regurgitation (AR).

**Methods:**

From May 2017 to August 2019, 16 patients (88% bicuspid aortic valves, no patients with connective tissue disorders) underwent aortic annuloplasty with an external complete Dacron band. Clinical and echocardiographic follow-up was 100% complete. Clinical and echocardiographic follow-up averaged 24.4 ± 9.3 and  15.1 ±  8.3 months, respectively.

**Results:**

Mean cardiopulmonary and cross-clamp times were 105 ± 15 (72–127) and 86 ± 15 (51–113) min, respectively. Early and late mortality was 0%, with no incidents of endocarditis or cerebrovascular events during the follow-up. Two patients were re-operated during the follow-up, one due recurrent aortic regurgitation (12 months after the first operation) yielding a freedom from reoperation due to AR at 1 year and 3 years of 100% ± 0% and 93.3% ± 5.7%, respectively. Based on the latest echocardiogram, five patients had either none or trivial AR, six had mild AR, and three had mild-to-moderate AR.

**Conclusions:**

The early clinical and echocardiographic results after using a complete external Dacron band are promising; however, more data and longer follow-up are needed to determine its role in annular management during aortic valve repair.

**Supplementary Information:**

The online version contains supplementary material available at 10.1007/s11748-021-01695-1.

## Introduction

Aortic valve repair has emerged as a surgical alternative to valve replacement in the setting of aortic regurgitation (AR) in selected patients [1]. Preserving the aortic valve has the potential to reduce some of the inherent limitations associated with prosthetic heart valves [2]. In analogy with the evolution of mitral valve repair, attempts have been made to standardize aortic valve repair to address the unique anatomy and function of the aortic root [3, 4]. Surgical treatment of AR in the setting of annular dilatation (type Ic lesion) or cusp prolapse (type II lesion) requires an annuloplasty approach, often in combination with cusp repair [3].

Several annuloplasty techniques have been described to reduce annular dilatation; external or internal suture annuloplasty, partial band annuloplasty, and complete rigid or flexible ring annuloplasty [5]. All these procedures aim to stabilize the aortic root, and thus enhance the long-term durability of the aortic valve repair. Lansac and colleagues have advocated the use of an external flexible ring annuloplasty at the subvalvular level to stabilize the aortic root over time [6]. Furthermore, Schäfers group in Homburg has used an external suture annuloplasty to improve the outcome of the aortic valve repair [7]. An alternative to a commercially available external flexible ring can be a self-made external band made from a Dacron graft. Thus, in type Ic or type II AR, we use a self-made complete external Dacron band to the reduce and stabilize the aortic annulus. The aim of this study was to report the early results after aortic annuloplasty with a complete external Dacron band.

## Materials and methods

### Study population

**Fig. 1:**
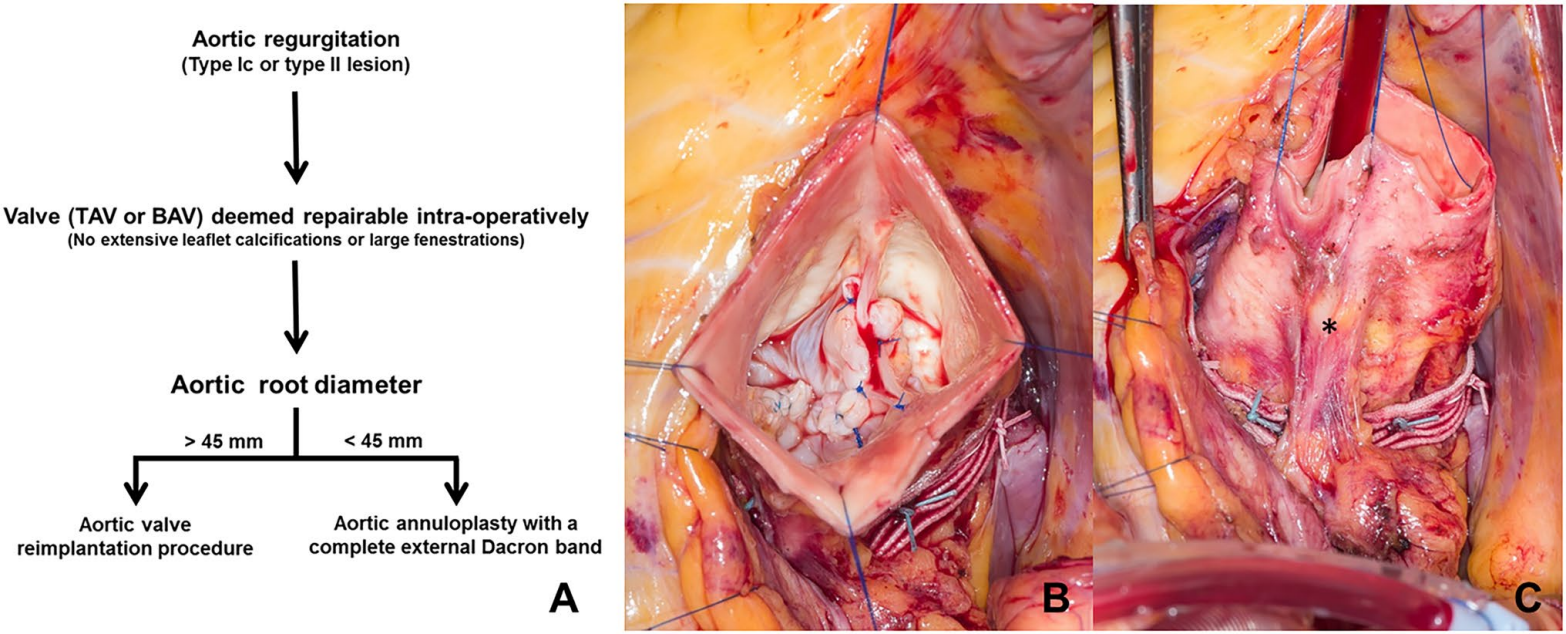
Simplified flowchart showing when we use an aortic valve reimplantation procedure or an aortic annuloplasty procedure with a complete external Dacron band in the setting of aortic valve repair [**A**]. Intra-operative picture of a resection of a fibrotic raphe and free margin cusp plications of both the fused and non-fused cusps and the external Dacron band in place [**B**]. Picture of the external Dacron band (the asterix marks the left main) [**C**]

The study was approved by the regional ethics committee at Oslo University Hospital (REK 2014/659), and written informed consent was obtained from all patients. Between May 2017 and July 2019, 16 consecutive patients (our initial experience) underwent aortic valve repair and annuloplasty using a complete external Dacron band (DuPont, Wilmington, DE, USA) at Oslo University Hospital, Rikshospitalet. Figure 1A shows a simplified flowchart for when we use an aortic valve reimplantation procedure or an aortic annuloplasty procedure with a complete external Dacron band in the setting of aortic valve repair. Detailed characteristics of the patients are listed in Table 1. We performed three procedures in 2017, seven in 2018, and six in 2019. The patient’s records were reviewed retrospectively. Clinical follow-up was censored on November 1 2020 and was 100% complete. Follow-up echocardiographic examinations were all performed at our institution. The pre-operative CT measurement of the ascending aorta (measured as the largest diameter in the ascendens) and the proximal aortic arch (measured between the innominate and the left common carotid artery) are listed in supplemental Table E1.

**Table 1:** Baseline clinical characteristics

Variables	
Age (y)	
Mean ± SD (range)	49 ± 11 (30–68)
Male gender, *N* (%)	14 (88)
Height (cm)	
Mean ± SD (range)	180 ± 7 (168–192)
Weight (kg)	
Mean ± SD (range)	86 ± 12 (72–110)
BMI (kg/m^2^)	
Mean ± SD (range)	27 ± 4 (23–35)
Connective tissue disorder, *N* (%)	0 (0)
Hypertension, *N* (%)	4 (25)
Previous cardiovascular surgery, *N* (%)	0 (0)
Tricuspid aortic valve, *N* (%)	2 (12)
Bicuspid valve, *N* (%)	14 (88)
Sievers type 1 L–R, *N* (%)	13 (93)
Sievers type 0, *N* (%)	1 (7)
Type A (symmetrical), *N* (%)	6 (43)
Type B (asymmetrical), *N* (%)	5 (36)
Type C (very asymmetrical), *N* (%)	3 (21)

*BMI* body mass index, *L–R* left–right, Type A (symmetrical BAV 160–180°),

Type B (asymmetrical BAV 140–159°), Type C (very asymmetrical BAV 120–139°)

### Surgical technique

The heart was exposed through a midline sternotomy and standard cardiopulmonary bypass was established. The ascending aorta was cross-clamped and the heart was arrested with antegrade cold crystalloid cardioplegia. The aorta was transected approximately 1 cm above the sino-tubular junction. The aortic valve was inspected and evaluated for any relative contraindications for repair such as extensive leaflet calcification or large fenestrations. Thereafter, the aortic root was dissected down to the level of the subvalvular plane. For the left and non-coronary sinuses, this was usually straightforward. The dissection of the right coronary sinus, however, was done down to the muscle of the right-ventricular outflow tract which limits the dissection in this area*.* The internal size of the aortic annulus was measured using a Hegar dilator. A Dacron graft of appropriate size was then selected and a circular part of the graft (i.e., the band) measuring 3–5 mm was cut off and transected. The length of the band, and thus the diameter of the neo-annulus, was dependent on body size. As a general rule, we aim the neo-annulus to be 22–24 mm in adult males and 1–2 mm less in smaller woman. Accordingly, a male with a 28 mm internal aortic annulus diameter should have an external band taken from a 28 mm Dacron graft to ensure a neo-annulus with an internal diameter of 23 mm.

The Dacron band was fixated in an external subannular position using 6–10 pledgeted U-polyester sutures (Ethibond Excel with TFE polymer pledgets 7 mm × 3 mm × 1.5 mm, Ethicon Inc, Sommerville, NJ, USA). The sutures were placed inside out circumferentially in the subvalvular plane 3 mm caudal to the nadir insertions of the leaflets. The exact number of sutures depends on the particular size and anatomy of the aortic root. One U-suture should be placed deeply on each side of the coronary arteries to ensure that the band does not slide and compress the arteries. Eventually, the upper part of the Dacron band can be cut beneath the artery after the band is in its exact position. We skip U-sutures in the left ventricle outflow tract under the commissure of the right/non to avoid damage to the conduction system.

The Dacron band was usually easy to thread under the right coronary artery. If the left main coronary artery was short, or it ran in parallel to and near the aortic wall, we interrupted the band in a length of 0.5–1 cm under the artery to avoid hazardous dissection or compression by the band. Finally, the U-sutures were tied and the Dacron band fixated over a Hegar dilator in left-ventricular outflow tract to ensure an adequate aortic annular diameter. Aortic valve repair was performed after the external band was in place, see Fig. 1B–C. Most of the downsizing of the external Dacron band was done along the fused cusps in the bicuspid valves, aiming for a final result with a commissural angle close to 180°. Intra-operative variables are summarized in Table 2.

**Table 2:** Intra-operative variables

Variables				
CPB time (min)				
Mean ± SD				105 ± 15 (72–127)
XC time (min)				
Mean ± SD				86 ± 15 (51–113)
Concomitant procedures				
Supracoronary graft, *N* (%)				4 (25)
Epicardial pacemaker leads, *N* (%)				1 (6)
Transposition of anomalous right coronary artery, *N* (%)				1 (6)
AV repair	BAV			TAV
	*Type A*	*Type B*	*Type C*	
	*n* = *6*	*n* = *5*	*n* = *3*	*n* = *2*
Free margin plication	6	5	3	2
Resection of raphe (direct closure)	3	2	2	0
Resection of raphe (bovine pericardial patch)	0	1	0	0
Pre-LVOT (Hegar, mm)				
Mean ± SD				29 ± 2 (25–36)
Neo-LVOT (Hegar, mm)				
Mean ± SD				22 ± 1 (21–25)

*CPB* cardiopulmonary bypass, *XC* cross-clamp, *AV* aortic valve repair, *BAV* bicuspid aortic valve, *TAV* tricuspid aortic valve, Type A (symmetrical BAV 160–180°), Type B (asymmetrical BAV 140–159°), Type C (very asymmetrical BAV 120–139°), *LVOT* left-ventricular outflow tract

### Clinical and echocardiographic follow-up

Post-operative valve-related adverse events were systematically registered and analyzed according to the American Association for Thoracic Surgery-Society of Thoracic Surgeons-European Association of Cardio-Thoracic Surgery guidelines for reporting morbidity and mortality after cardiac valve interventions [8]. The average time for clinical follow-up was 24.4 ± 9.3 months (median 21 months, interquartile range 17–32 months; maximum 41 months). We obtained transthoracic echocardiograms on all patients. The average time for the latest transthoracic echocardiogram was  15.1 ±  8.3 months (median 13.5 months; interquartile range  11.5–17 months; maximum 32 months).

### Statistical analysis

Data were retrospectively collected, analyzed, and visualized using GraphPad Prism 8.0.1 for Windows (GraphPad Software, La Jolla, CA, USA). The Shapiro–Wilk test was used to test if continuous variables were normally distributed. Comparison of continuous variables before and after surgery was done using the Student t-test if normally distributed or the Mann–Whitney test if not normally distributed. Continuous variables are presented as mean ± standard deviation or median and interquartile range (25–75%) depending on their normality distribution and dichotomous variables are presented as percentages. A probability value of less than 0.05 was considered statistically significant. The Kaplan–Meier survival estimate was used to analyze freedom from reoperation due to recurrent AR.

## Results

### Survival and reoperations

**Fig. 2:**
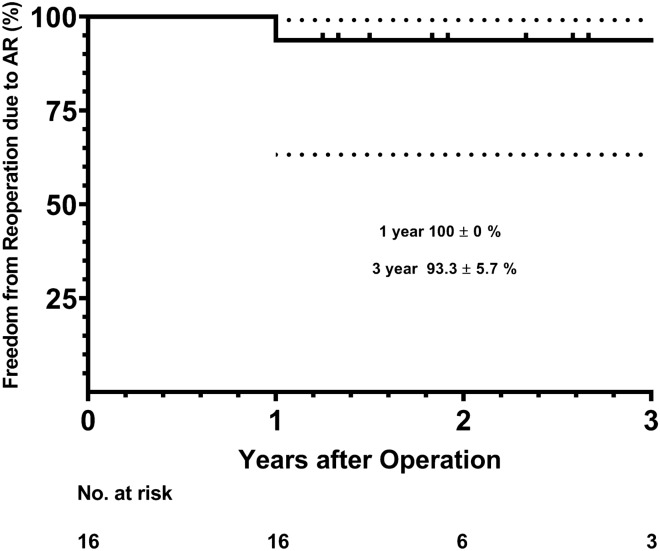
Freedom from reoperation due to aortic regurgitation (AR) after aortic annuloplasty using an external Dacron band. Dotted lines represent 95% confidence intervals

Early and late mortality was 0%. We had one patient where the band caused kinking of the circumflex artery that was identified intra-operatively, so the band was removed under the left main coronary artery. The patient had higher levels of cardiac enzymes the first 2 post-operative days, but otherwise had an uneventful recovery. No patient was re-operated due to bleeding, developed renal failure or mediastinitis, or needed a permanent pacemaker. The median length of stay was 4.5 days (IQR 4–6.25). One patient required reoperation due to recurrent AR due to prolapse of the fused coronary cusps, 12 months after the first operation. Furthermore, one patient was re-operated 15 months after the first operation due to kinking of an anomalous right coronary artery that was transposed at the time for the aortic valve repair surgery. The freedom from reoperation due to recurrent AR at 1 and 3 years was 100% ± 0% and 93.3% ± 5.7%, respectively (Fig. 2). No incidents of cerebrovascular events or endocarditis have been observed during the follow-up. Furthermore, during the follow-up, we had no events of aortic dissection or band migration. All patients have done a post-operative CT scan approximately 3 months after the surgery and we have no case of pseudoaneurysme in the proximal or distal suture line.

### Aortic valve repair

In addition to the annuloplasty, all patients required an aortic valve cusp repair. The types of aortic valve repair stratified according to the commissural orientation of the bicuspid aortic valve are summarized in Table 2. The median aortic annulus measured with Hegar dilator was significantly reduced (*p* < 0.01) after the annuloplasty. Mean native and neo-annulus diameters were 29 ± 2 mm (25–36 mm) and 22 ± 1 mm (21–25 mm), respectively. In supplemental Table E2, the size of the Dacron band is listed with the corresponding pre-operative and neo-left-ventricular outflow tract measured using Hegar dilators. Preoperatively, 15 of 16 patients had an eccentric AR, while only 4 had an eccentric AR on follow-up. Eleven patients had a central AR on follow-up. The antegrade mean gradient after the procedure was 14 ± 7 mmHg (5–31 mmHg) as compared to 10 ± 4 mmHg (2–17 mmHg) at baseline (p =  0.11).

### Aortic valve function, left ventricular, and aortic root size based on echo

**Fig. 3:**
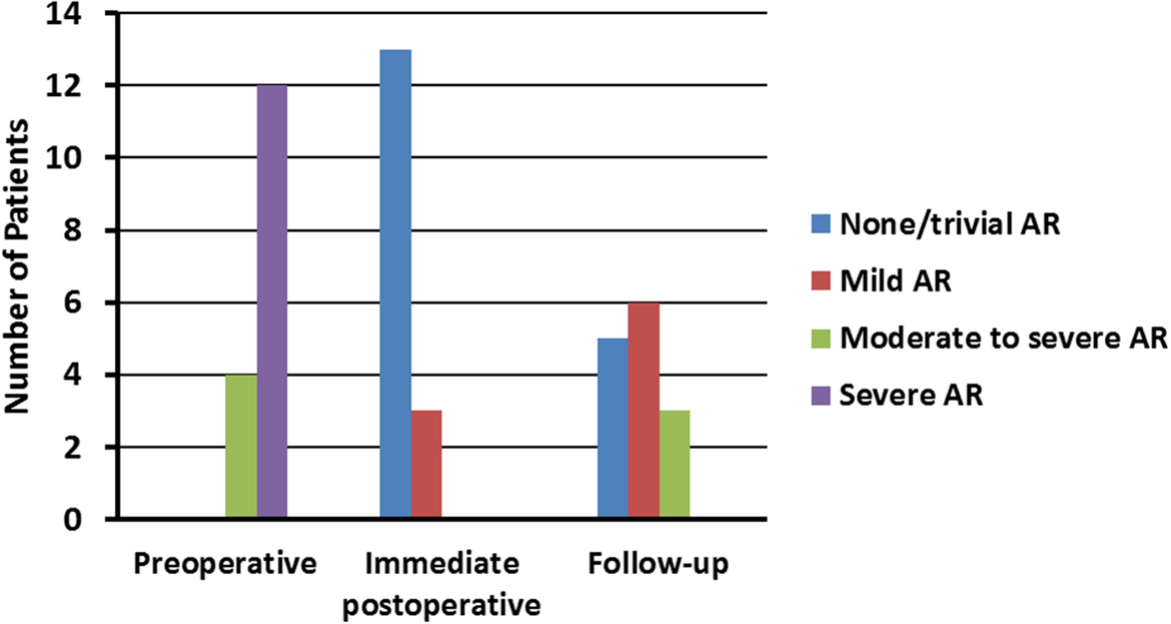
Aortic regurgitation grade pre-operative, immediate post-operative, and at follow-up. *AR* aortic regurgitation

Based on the latest echocardiogram,  five patients had either none or trivial AR,  six had mild AR, and  three had mild-to-moderate AR (Fig. 3). Comparing pre-operative and follow-up echocardiograms, we found a significant reduction in left-ventricular end-diastolic and end-systolic diameters after the annuloplasty procedure (Table 3). The coaptation length was significantly (*p* < 0.01) greater after the operation, with a pre-operative and follow-up lengths of 3 ± 3 mm (0–8 mm) and 7 ± 3 mm (4–11 mm), respectively. Furthermore, the effective height also increased significantly (*p* < 0.01) from 3 ± 3 mm (0–8 mm) preoperatively to 9 ± 4 mm (0–13 mm) at follow-up. The follow-up value of the post-operative annulus was significantly larger than the immediate annulus values, 25 ± 2 mm (20–29) vs. 23 ± 1 mm (20–26), respectively (*p* = 0.02). The size of the aortic annulus was significantly reduced after the annuloplasty, while the sinuses of Valsalva and the sino-tubular junction remained the same. Left-ventricular ejection fraction remained unchanged from pre-operative to follow-up echocardiograms (Table 3).

**Table 3:** Echocardiographic data

	Preoperative	Immediate post-operative	Follow-up	*P* value ✝
Variables*				
LVEDD (mm)	67 ± 7 (55–77)	57 ± 7 (45–68)	60 ± 6 (52–73)	< 0.01
LVESD (mm)	48 ± 8 (29–65)	43 ± 6 (45–68)	43 ± 7 (34–60)	0.02
LVEF (%)	53 ± 7 (40- 65)	47 ± 6 (30–60)	52 ± 6 (40–60)	0.99
AR grade, *N* (%)				
None/trivial	0 (0)	13 (81)	5 (36)	
Mild	0 (0)	3 (19)	6 (43)	
Moderate-to-severe	4 (25)	0 (0)	3 (21)	
Severe	12 (75)	0 (0)	0 (0)	
Mean (± SD)	2.8 ± 0.3	0.4 ± 0.3	1.0 ± 0.7	
Central AR	8	10	11	
Eccentric AR	15	1	4	
Antegrade gradient (mmHg)	10 ± 4 (2–17)	12 ± 5 (1–21)	14 ± 7 (5–31)	0.11
Effective height (mm)	3 ± 3 (0–8)	8 ± 3 (0–12)	9 ± 4 (0–13)	< 0.01
Coaptation length (mm)	3 ± 3 (0–8)	7 ± 2 (4–10)	7 ± 3 (4–11)	< 0.01
Annulus (mm)	29 ± 3 (25–35)	23 ± 1 (20–26)	25 ± 2 (20–29)	< 0.01
Sinus (mm)	39 ± 4 (30–44)	36 ± 5 (30–45)	38 ± 4 (30 -44)	0.58
STJ (mm)	33 ± 4 (30–44)	31 ± 3 (26–36)	32 ± 3 (28–38)	0.24

Data are presented as mean ± SD (range). ✝The Shapiro–Wilk test was used to test if continuous variables were normally distributed. Comparison of continuous variables before and after surgery was done using the Student  t-test if normally distributed or the Mann–Whitney test if not normally distributed. *LVEDD*, left-ventricular end-diastolic diameter, *LVESD* left-ventricular end-systolic diameter, *LVEF* left-ventricular ejection fraction, *AR* aortic regurgitation,*STJ* sino-tubular junction

## Discussion

In this study, we found good early results after aortic annuloplasty using a complete external Dacron band in patients with type Ic or type II AR. There is much controversy on how to perform an annuloplasty procedure in the setting of aortic valve repair to stabilize the aortic annulus over time [5, 9]. In the literature, a number of methods have been described, ranging from external or internal suture annuloplasty, partial band annuloplasty, and ring annuloplasty with rigid or flexible rings [5, 9].

A large body of data on the external flexible ring annuloplasty implanted at the subvalvular level has been reported in the literature, and the long-term outcome of this method is satisfactory for both bicuspid and tricuspid valves [10]. As presented herein, an alternative to a commercially available annuloplasty ring can be a self-made external band made from a Dacron graft. Currently, however, we have no data comparing the two methods of annuloplasty with each other, so they might not be equivalent in terms of durability of the aortic valve repair.

Our approach to aortic annuloplasty, using an external Dacron band, resembles the operation described by David in 1996 where he combined root remodeling with band annuloplasty [11]. In the excellent review by Kunihara on annular management during aortic valve repair, the outcomes after a partial band annuloplasty are limited to data reported by David and Song and colleagues from Korea [5]. In the data from Toronto, the partial band annuloplasty is used in patients undergoing aortic remodeling, so the outcome of the annuloplasty per se is unclear [11]. The data from Korea are associated with high 1-year cumulative incidences of major bleeding, reoperation, endocarditis, and death (3.55%, 5.65%, 5.05%, and 5.33% per year, respectively) [13]. These data, however, are not in patients with only an external partial annuloplasty band and cusp repair. In the original report by Hahm and colleagues, they describe extensive surgical procedures in patients with and without aortic root disease using an internal synthetic strip and external partial or complete ring as well as extensive aortic valve repair including additional leaflets for leaflet correction [14]. Therefore, these data cannot support the conclusion that long-term outcome after external band annuloplasty band is inferior to other techniques aiming to stabilize the aortic annulus.

In connective tissue disease, bicuspid aortic valves, and idiopathic annuloaortic ectasia, annular dilatation is a major component of the aortic root pathology. David and Shah state that more of the dilatation takes place along the fibrous part of the annulus from the membranous septum along the top of the mitral to the left trigonum [11, 12]. They therefore argue that a partial band along this fibrous part of the annulus would be sufficient to stabilize the aortic root. Data supporting such pathology and surgical solution, however, are not conclusive. Kim et al*.* has shown using 3-dimensional cardiac computer tomography that dilatation of the aortic root is even more pronounced in the right and non-coronary parts of the circumference [15]. Similarly, in mitral regurgitation, the dilatation is found predominantly in the posterior, muscular part, not in the aortomitral skeleton. To ensure that the whole aortic annulus is stabilized over time, we therefore implant a complete, circular band. The band is usually easy to thread under the right coronary artery, so a circular band does not significantly increase the complexity of the procedure compared to a partial band. We do not place U-sutures in the left-ventricular outflow tract to avoid damage to the conduction system. We had no patient that needed a permanent pacemaker in our cohort. Surgeons familiar with valve-sparing aortic root procedures can easily adopt this annuloplasty technique as the external dissection of the aortic root down to the ventricular junction is the same. We have no patient with connective tissue disorder in our cohort, but we do not consider this a contraindication to the procedure, since intra-operative findings of the valve determine if the valve is suitable for repair. On the other hand, most patients with connective tissue disorder and type Ib AR will be treated with aortic valve reimplantation.

In our cohort, we had patients with type Ic and type II AR. All 16 patients underwent aortic valve repair with free margin plication for the correction of cusp prolapse similar to the technique described by Boodhwani and colleagues [16]. Furthermore, 7 patients underwent resection of the fibrotic or calcified raphe in addition to the free margin plication.

A proper understanding of aortic root anatomy and physiology is fundamental to further advance and refine aortic valve repair [17]. For the external annuloplasty procedure, a complete dissection of the proximal aortic root is mandatory. The aortoventricular junction is not planar and it is therefore important to secure the graft, band, or ring in a subvalvular position [4].

In the setting of aortic root dilation, proponents of the remodeling technique have advocated that such an approach preserves the physiological function of the aortic valve in contrast to the aortic valve reimplantation procedure. To ensure that the aortic root does not continue to dilate, an external subvalvular annuloplasty ring is often used in adjunction with root remodeling with good long-term results [18]. Whether such a ring ensures the physiological function of the aortic valve over time remains to be confirmed. In the setting of mitral valve surgery, Bothe and colleagues have clearly demonstrated in an acute ovine model that a partial band as well as a semi-rigid or a rigid annuloplasty ring obliterates the movement of the mitral annulus throughout the cardiac cycle [19].

The excellent long-term data using the aortic valve reimplantation technique, both in the setting of connective tissue disorders as well as tricuspid and bicuspid aortic valves, emphasize the importance of fixating the subvalvular aortic root to prevent future dilatation [20, 21]. In our patient cohort, we had no patient with connective tissue disorder, but 88% of the patients had a bicuspid aortic valve. According to the classification of bicuspid valves proposed by Sievers, we had 13 type 1 L–R valves and one valve with a type 0 configuration [22].

Medium- and long-term results of aortic valve reimplantation procedure in the setting of bicuspid aortic valves are acceptable, and systematic assessment of effective cusp height and stabilization of the aortic root in some form is associated with improve durability of the repair [23]. None of the patients in the current cohort had an aortic root > 45 mm and therefore were not in our practice candidates for aortic valve reimplantation procedure (Fig. 1A). Since 2004 we have done more than 200 aortic valve reimplantation procedures at our institution, and deemed the patients in the current cohort not to fulfill the criteria for a reimplantation procedure.

The natural history of bicuspid aortic valves is associated with progressive valve dysfunction, and many of our patients will most likely require a secondary intervention after their first operation [24]. Data suggest that higher gradients after aortic valve repair are associated with reduced freedom from re-intervention [25]. We registered a modest increase after aortic valve repair from mean gradient of 10 mmHg at baseline to 14 mmHg at follow-up. It is not likely that this translates into adverse clinical results.

Some recent data indicate that aortic valve repair with ring annuloplasty both at the subvalvular level as well as the sino-tubular junction is associated with improved outcome compared to only a ring at the subvalvular level [26]. In our cohort, 4 out of 16 patients had a dilated ascending aorta requiring a concomitant supracoronary graft (the pre-operative CT measurements of the proximal aorta in our patients are listed as supplemental Table E1).

## Limitations

There are several important limitations associated with the data presented herein. The small number of patients, the short follow-up, and that all procedures were performed by one surgeon (R.L.) makes generalization of the intra-operative data as well as outcome difficult. We had consecutive cases and 100% complete clinical and echocardiographic data making the results reliable and transparent.

In conclusions, the early results after using a complete external Dacron band are promising; however, more data and longer follow-up are needed to determine its role in annular management during aortic valve repair.

## Supplementary Information

Below is the link to the electronic supplementary material.

Supplementary file1 (DOCX 19 KB)

Supplementary file2 (DOCX 22 KB)
