# Supplementary material for: Early results after aortic annuloplasty with a complete external Dacron band
Source: Gen Thorac Cardiovasc Surg. 2021 Sep 20;70(4):329–36. doi: 10.1007/s11748-021-01695-1 (PMC8930909; doi:10.1007/s11748-021-01695-1)
Supplement: Supplementary file 1 — Supplementary file1 (DOCX 19 KB) [file 11748_2021_1695_MOESM1_ESM.docx]

Table E1: Pre-operative CT measurements of the proximal aorta

Patient # Aorta Ascendens Proximal aortic arch

* 1. 45 25

* 2. 53 31

3. 42 25

4. 42 23

5. 41 26

6. 28 21

7. 38 24

* 8. 46 25

9. 40 28

10. 38 26

11. 31 22

12. 43 28

13. 44 28

14. 33 25

15. 38 25

* 16. 55 26

Mean ± SD 41 ± 7 26 ± 2

* Patients that had concomitant replacement of the ascending aorta with a supracoronary graft. Ascending aorta measured as the largest diameter in the ascendens. Proximal arch measured between the innominate and the left common carotid artery.
