# Supplementary material for: Early results after aortic annuloplasty with a complete external Dacron band
Source: Gen Thorac Cardiovasc Surg. 2021 Sep 20;70(4):329–36. doi: 10.1007/s11748-021-01695-1 (PMC8930909; doi:10.1007/s11748-021-01695-1)
Supplement: Supplementary file 2 — Supplementary file2 (DOCX 22 KB) [file 11748_2021_1695_MOESM2_ESM.docx]

Table E2: Relationship between the Dacron graft and Hegar measurements of LVOT

Patient # Pre LVOT (Hegar, mm) Neo LVOT (Hegar, mm) Dacron graft (mm)

1. 28 23 30

2. 30 24 30

3. 31 23 28

4. 28 23 28

5. 27 21 28

6. 25 21 28

7. 32 22 30

8. 30 22 28

9. 30 21 28

10. 29 21 28

11. 29 21 28

12. 29 22 28

13. 36 25 30

14. 28 22 28

15. 28 23 28

16. 28 22 28

Mean ± SD 29 ± 2 (25-36) 22 ± 1 (21-25) 29 ± 1 (28-30)

LVOT, left ventricular outflow tract.
